# Technical considerations and long-term outcomes of successful one-stage repair of Berry syndrome in a preterm neonate using deep hypothermic circulatory arrest: A case report

**DOI:** 10.1016/j.ijscr.2025.111749

**Published:** 2025-07-29

**Authors:** Guangguo Men, Junxian Chen, Beirong Yu

**Affiliations:** a Women and Children's Hospital of Ningbo University, Ningbo, Zhejiang, China

**Keywords:** Berry syndrome, Congenital heart disease, Preterm neonate, Surgical repair

## Abstract

**Introduction and importance:**

Berry syndrome is a rare congenital cardiac anomaly characterized by aortopulmonary window, aortic origin of the right pulmonary artery, interrupted aortic arch, interrupted aortic arch or hypoplastic aortic arch or coarctation of the aorta, and intact ventricular septum.

**Case presentation:**

We report a case of a preterm neonate with Berry syndrome who underwent successful surgical correction. The patient, a 34^+4^ weeks gestational age male, was diagnosed prenatally with complex congenital heart disease and genetic abnormalities. Postnatal echocardiography confirmed Berry syndrome. Surgical intervention was performed on the fourth day of life, involving deep hypothermic circulatory arrest for aortic window repair, ductal ligation, and aortic arch reconstruction.

**Clinical discussion:**

Berry syndrome poses significant challenges due to its complex anatomy and the need for early surgical intervention. The use of deep hypothermic circulatory arrest allows for a bloodless field and precise repair of the cardiac anomalies. Prolonged mechanical ventilation and delayed sternal closure are often necessary in preterm neonates due to their compromised cardiopulmonary reserve.

**Conclusion:**

Early diagnosis and timely surgical intervention are crucial for the survival of neonates with Berry syndrome.

## Highlights

• First report of successful Berry syndrome repair in a preterm neonate (34^+4^ weeks GA)
• Definitive single-stage surgical correction performed on day 4 of life using deep hypothermic circulatory arrest.
• Surgical techniques included aortopulmonary window patch closure, PDA ligation, and aortic arch reconstruction.
• Multidisciplinary team management and delayed sternal closure were crucial for successful outcome despite prematurity.
• Case adds to limited literature, demonstrating favorable results possible with early diagnosis and meticulous intervention.

## 1 Introduction

Berry syndrome is among the rarest of congenital cardiac malformations. Since it was firstly described by Berry et al. [1], barely 100 cases have been reported worldwide. The lesion is defined by a pathognomonic quartet of findings: an aortopulmonary window, anomalous aortic origin of the right pulmonary artery, interrupted aortic arch (almost always type A), and an intact ventricular septum [2]. A patent ductus arteriosus co-exists in >98 % of patients [3], rendering the circulation ductal-dependent and acutely life-threatening once spontaneous ductal closure begins.

Epidemiological data remain sparse. The best contemporary estimate places the incidence at <1 per 1,000,000 live births, and only a handful of affected infants have been born before 35 weeks' gestation. Consequently, most knowledge is extrapolated from isolated truncal or arch anomalies; evidence-based algorithms for preterm neonates are essentially absent.

The therapeutic imperative is unequivocal: without early surgical repair, progressive heart failure and systemic hypoperfusion are almost uniformly fatal within the first weeks of life. Over the past two decades, A comprehensive one-stage repair surgery which consists of patch closure of the aortopulmonary window, ligation of the arterial duct, and reconstruction of the interrupted arch-has supplanted staged palliation, yielding superior survival rates of 85.7 % in infants [4]. However, these outcomes derive from cohorts with median birth weights >2.5 kg and gestational ages >37 weeks.

Preterm neonates introduce a constellation of additional challenges: immature myocardium with diminished calcium-handling reserve, surfactant-deficient lungs prone to barotrauma, fragile cerebral vasculature susceptible to hypothermic circulatory arrest–related injury, and limited vascular access which complicates cardiopulmonary bypass. Moreover, the diminutive ascending aorta (often <5 mm) necessitates technically demanding patch augmentation during periods of low-flow or circulatory arrest, while post-operative fluid balance must be managed within a narrow margin to avoid both systemic edema and inadequate preload.

We therefore report the successful single-stage repair of Berry syndrome in a 34^+4^-week, 2000 g neonate operated on day 4 of life. Beyond detailing the surgical technique, we emphasize that the multidisciplinary perioperative strategy should combine with prenatal diagnosis, modified ultrafiltration, neuroprotective hypothermia, and restrictive fluid protocols-that enabled survival without major morbidity. To our knowledge, this is the first documented case of such early definitive correction in a preterm infant, providing new evidence that excellent outcomes are attainable even at the limits of viability.

This case report has been reported in line with the SCARE checklist [5].

## 2 Case presentation

**Fig. 1:**
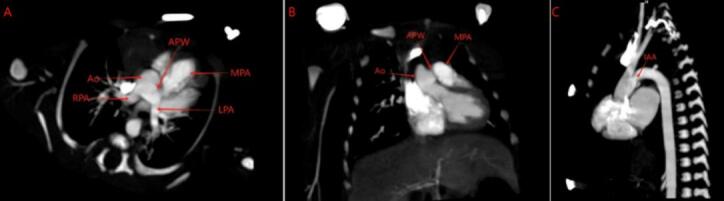
Computed tomography angiography (CTA) demonstrated type II aortopulmonary window (APW). The right pulmonary artery (RPA) arose from the ascending aorta (Ao). The left pulmonary artery (LPA) had normal origin from the main pulmonary artery (MPA). The aortic arch was interrupted distal to the left subclavian artery (IAA).

A male neonate was born at 34^+4^ weeks' gestational age via cesarean section due to maternal complications including placenta previa, placenta accreta, scarred uterus, and threatened preterm labor. The birth weight was 2000 g, with Apgar scores of 9 at both 1 and 5 min. Postnatal echocardiography revealed right pulmonary artery arising anomalously from the aorta, aortopulmonary window, interrupted aortic arch (Type A), and patent ductus arteriosus, confirming the diagnosis of Berry syndrome (Fig. 1). The patient's parents agreed to the surgical plan we provided and signed an informed consent form.

On the fourth day of life, the neonate underwent surgical correction under deep hypothermic circulatory arrest. The procedure involved repair of the aortopulmonary window using a pericardial patch, ligation of the patent ductus arteriosus, and reconstruction of the interrupted aortic arch. The surgery was performed via a median sternotomy, with cardiopulmonary bypass established via aortic and right atrial cannulation. The aortic arch was reconstructed by directly anastomosing the posterior wall and patch augmentation of the anterior wall using pericardium. After adequate exposure, the aortic arch and descending aorta were dissected, confirming a type A aortic interruption. Core temperature was reduced to 25 °C, the aorta was cross-clamped, and cold blood cardioplegia was delivered antegrade. When the nasopharyngeal temperature reached 22.4 °C, cardiopulmonary bypass was temporarily discontinued. The hypoplastic ascending aorta was longitudinally incised. A large aortopulmonary window, approximately 10 mm in diameter, was identified, along with the origins of the left and right pulmonary arteries. A fresh autologous pericardial patch was fashioned to close the aortopulmonary window, effectively baffling the branch pulmonary arteries into the main pulmonary trunk. The arterial duct was ligated at its pulmonary end and divided; the ductal tissue was completely excised. The distal descending aorta was mobilized and brought cephalad. The posterior wall of the descending aorta was anastomosed directly to the undersurface of the distal aortic arch in an end-to-side fashion. The anterior circumference of this anastomosis was augmented with a second, carefully tailored pericardial patch to enlarge the reconstructed aortic arch and restore unobstructed continuity. After completion of the repair, air was evacuated, cardiopulmonary bypass was reinstituted, and the patient was gradually rewarmed. Hemostasis was secured, and the patient was transferred to the intensive care unit in stable condition. Due to hemodynamic instability, delayed sternal closure was employed. The aortic cross-clamp time was 59 min, and the total cardiopulmonary bypass time was 100 min.

The patient required mechanical ventilation for 8 days postoperatively. Intensive care management included inotropic support by intravenous infusion of dobutamine, controlling the liquid intake to approximately 150 ml per kilogram of body weight per day to achieve a balanced intake and output. Due to the patient's pneumonia, we discontinued the use of antibiotics vancomycin and piperacillin/sulbactam after 6 days. Gradually, weaning from respiratory support was achieved, and the patient showed significant improvement in cardiac function. Follow-up echocardiography demonstrated satisfactory repair with no residual shunts or obstructions.

Genetic testing revealed a heterozygous missense variant c.152G > C (p.Arg51Pro) in the Sox11 gene of the tested individual. Second-generation and first-generation sequencing results showed that neither of the tested individual's parents carried this variant. It is speculated that this variant in the tested individual is a de novo mutation or that one of the parents has germline mosaicism.

The patient was hospitalized for a total of 41 days and was discharged 37 days after surgery. No postoperative complications were observed. Six-month follow-up has shown that the child's growth and neurological development are normal, and the cardiac ultrasound results only indicate a patent foramen ovale of 4.3 mm. Since the follow-up, the parents of the child have expressed satisfaction with the treatment.

## 3 Clinical discussion

Berry syndrome represents a critical congenital cardiac anomaly demanding urgent surgical intervention [6,7]. This case report is notable for describing the successful single-stage repair in a preterm neonate (34^+4^ weeks gestational) – a population with inherently heightened surgical risks due to cardiopulmonary immaturity and low birth weight (2000 g). Our experience underscores that early diagnosis (prenatal suspicion confirmed postnatally) and definitive surgery on the 4th day of life are paramount for survival, preventing ductal-dependent circulation collapse and progressive heart failure.

The surgical strategy employed deep hypothermic circulatory arrest, which was essential for achieving a bloodless field and facilitating precise reconstruction of complex anomalies: aortopulmonary window closure using an autologous pericardial patch, ligation of the patent ductus arteriosus, and aortic arch reconstruction via direct anastomosis with anterior patch augmentation. Deep hypothermic circulatory arrest, while carrying inherent neurological risks, was indispensable given the intricate anatomy and the neonate's small thoracic cavity. The necessity for delayed sternal closure due to hemodynamic instability and the requirement for prolonged mechanical ventilation (8 days). These challenges reflect the compromised cardiopulmonary reserve in this vulnerable cohort, demanding meticulous postoperative management including inotropic support and strict fluid balance.

Paul MH showed that Sox11 is required for proper development in both mesodermal cells and neural crest cells. Deletion in either mesoderm or neural crest, or both, leads to outflow tract defects ranging from double outlet right ventricle to common arterial trunk [8].

Multidisciplinary coordination involving neonatology, pediatric cardiology, cardiac surgery, and intensive care was instrumental in navigating perioperative complexities. The identification of a de novo heterozygous Sox11 missense variant c.152G > C (p.Arg51Pro) adds a novel dimension, suggesting potential genetic associations warranting further investigation, while its direct pathogenicity in Berry syndrome remains unconfirmed. While favorable outcomes are achievable, as demonstrated by satisfactory follow-up echocardiography and clinical recovery, this report acknowledges the limitations inherent to a single case.

## 4 Conclusion

Early diagnosis and timely surgical intervention are crucial for the survival of neonates with Berry syndrome. Despite the complexity of the condition and the associated risks, successful outcomes can be achieved with meticulous perioperative management. This case adds to the limited literature on Berry syndrome and highlights the potential for favorable results even in preterm neonates.

## Author contribution

Guangguo Men conceptualized and designed the study, drafted the initial manuscript, and critically reviewed and revised the manuscript. Junxian Chen and Beirong Yu conceptualized and designed the study, and critically reviewed and revised the manuscript for important intellectual content.

## Consent

Written informed consent was obtained from the patient's parents for publication and any accompanying images. A copy of the written consent is available for review by the Editor-in-Chief of this journal on request.

## Ethical approval

Ethical approval for this study (No. NBFE-2025-KY-085) was provided by the Ethical Committee of Women and Children's Hospital of Ningbo University, Ningbo, China on 21 April 2025.

## Guarantor

Guangguo Men, Junxian Chen and Beirong Yu.

## Research registration number

Not applicable.

## Funding

This work was supported by the Medical Health Science and Technology Project of Zhejiang Province (No. 2021KY1053), the Ningbo Science and Technology Project (No. 2017C50052), and the Innovation Project of Distinguished Medical Team in Ningbo (No. 2022020405).

## Conflict of interest statement

The authors have no conflicts of interest relevant to this article to disclose.
